# Acknowledgement to Reviewers of *Nutrients* in 2013

**DOI:** 10.3390/nu6030922

**Published:** 2014-02-28

**Authors:** Nutrients Editorial Office

**Affiliations:** MDPI AG, Klybeckstrasse 64, CH-4057 Basel, Switzerland

The editors of *Nutrients* would like to express their sincere gratitude to the following reviewers for assessing manuscripts in 2013:
Abadie, ValérieAnuradha, CvBarnig, CindyAbate, NicolaArcot, JayashriBartlett, HannahAbbey, ElizabethArendt, Lisa M.Barvencik, FlorianAbd Rashed, AswirAreta, JoseBaumgartner, JeannineAbsah, ImadAruoma, OkezieBays, Harold E.Ackroff, KarenAugustin, LiviaBeatty, StephenAgostoni, CarloAveleira, CéliaBeck, KathrynAhmad, NihalAzqueta, AmayaBeighton, DavidAigner, ElmarAzzini, E.Beirne, RaymondAisen, PhilipBaba, E.Bell, DavidAl-Dujaili, EmadBacchetti, TizianaBelluci, Marina M.Alaedini, ArminBacon, CjBergman, PeterAlexander, Jonathon S.Bae, Eun HuiBernstein, AdamAlghannam, Abdullah F.Baidoo, SamBertrand, PaulAli, A.Bair, Ming-JongBetancor, Mónica B.Ali, Declan W.Baker, JulienBhattacharya, ArupAliberti, JulioBaker-French, SophiaBhattacharya, RajibAlvarez-Velilla, RaquelBaker, Lindsay B.Biagi, FedericoAmarowicz, RyszardBakker, SjoerdBibus, DouglasAncheta, IrmaBanerjee, SanjayBielohuby, MaximilianAnderson, G. HarveyBansal, Sukhvinder S.Biesalski, Hans KonradAnderson, JohnBarbagallo, MarioBiltagi, Mohammed AlAndres, AndrèsBarker II, Felix M.Bishop, PhilAngelico, FrancescoBarker, TylerBjörklund, AnneliAnshel Od, JeffBarnard, Neal D.Blumberg, JeffreyAntonogeorgos, GeorgeBarnes, Matthew J.Blundo, C.Bocarsly, MiriamCaldwell, Kathleen L.Ciclitira, PaulBohn, TorstenCamargo, CarlosClegg, DeborahBonaccorsi, GuglielmoCamilleri, MichaelClugston, RobinBondonno, CathyCampisi, JayCogswell, MaryBooth, SarahCampos, Dr. Michael A.Collins, AndrewBorchhardt, KyraCampoy, CristinaColon-Ramos, UriyoanBorén, JanCañas, B.Conigrave, ArthurBorghi, ElaineCanene-Adams, KirstieConsidine, Robert V.Bortolus, RenataCantorna, MargheritaConti, ArioBosch, GuidoCao, Jay J.Cook-Mills, Joan M.Botham, KathyCapellas, MartaCoppola, GiangennaroBoulet, Louis-PhilippeCaroli, A.Corazza, Gino RobertoBoy, ErickCasto, BruceCornwall, M. CarterBozzetti, FedericoCatassi, CarloCostabile, AdeleBrabet, PhilippeCatto-Smith, AnthonyCraig, Stuart AsBradbury, JoanneCecílio, Alzira BatistaCravo, MaríliaBraidy, NadyCermak, R.Crawley, HelenBramley, PeterCervera, C.Crei, FranzcogBrantly, MarkChadha, ManojCroese, JohnBranum, Amy M.Chan, MariaCross, CarrollBravenboer, NathalieChan, MarkCrozier, Sarah R.Briley, MargaretChan, RuthCulebras, JesusBrindal, EmilyChandra, Lawrance C.Cupisti, AdamascoBrock, KayeChang, Hsueh-WeiCureton, Pamela A.Brown, Amy C.Chang, Jer-MingCurran, ChristineBrown, Andrew W.Chaucheyras-Durand, F.Cusack, SiobhanBrown, LindsayChen, Cheng-ShengD’Archivio, MassimoBrown, RachelChen, Thomas C.Dallinga-Thie, Geesje M.Brownlee, IainCheng, H. L.Dangour, AlanBruick, Richard K.Cherbuin, NicolasDarewicz, MałgorzataBrumaghim, J.L.Cherney, DaveDarling, PaulineBucchianeri, MichaelaChesney, Russell W.Das, U.n.Buddington, Randal K.Chhagan, Meera K.Davids, MariskaBurk, RaymondChiavaroli, ValentinaDavis, CindyBurke, LoraChilds, CarolineDay, AndrewButterick, TammyChilibeck, PhilDe Araujo, IvanButterweck, VeronikaChirumbolo, SalvatoreDe Backer, GuyButzner, J. D.Choi, Kyung-HyunDe Bree, EelcoByrn, MaryChristopher, KennethDe Magistris, LauraCalder, PhilipChua, Anita C. G.De Smet, StefaanCalderon, LilianChun, OckDe Souza, RussellCaldovic, LjubicaCibulskis, CatherineDecker, Eric A.Decsi, TamasErkkola, MaijaliisaGadella, Bart M.Dehghan, MahshidEsteban, Maria ÁngelesGailer, JürgenDel Pino, JavierEstruch, RamónGaivão, IsabelDel Rio, DanieleEvans, Robert W.Galvano, FabioDelgado-Noguera, Mario F.Eyles, DarrylGambini, JuanDepalma, Ralph G.Eyres, LaurenceGarbis, SpirosDerbyshire, E.J.Failla, Mark L.Garcia-Larsen, VanessaDeut, Nicolaas E.P.Falk, MichaelGarcía-Marcos, LuisDeVito, ElisabettaFardet, AnthonyGarcia-Rios, AntonioDhillon, Karaj S.Farías, JorgeGarcia-Ruiz, Garcia-RuizDhillon, VarinderpalFaulconbridge, Lucy F.Garcia, DiegoDi Iorio, BiagioFayad, RajaGarland, CedricDickey, WilliamFeighery, ConlethGarner, BrettDideriksen, KasperFelin Scalabrin, Deolinda M.Geber, JonnyDierkes, JuttaFernandez, Maria LuzGeelen, AnoukDjuric, ZoraFerrero, DarioGeha, PaulDoig, GordonFerri, ClaudioGenuis, Stephen J.Dong, YuqingFesti, DavideGietl, EvaDonini, LorenzomariaField, CatherineGilbert, C. R.Donnelly, AlanFinck, Brian N.Gill, MaggieDowning, JeffreyFiorucci, StefanoGillum, Trevor L.Doyle, ToddFoo, Leng HuatGiovannucci, EdwardDraijer, RichardFord, DianneGiudetti, AnnaDrevon, Christian A.Fossen, TorgilsGleeson, MikeDrossopoulou, Garyfalia I.Foster, CharlesGoedecke, JuliaDrummond, Micah J.Francavilla, RuggieroGohlke, HelmutDumke, Charles L.Francesco, MasoeroGonzález-Stuart, ArmandoDunkov, BorisFrancki, M. G.Gorham, EdwardDunn, WilliamFranz, MarionGotoda, TakanariDunstan, ColinFranzese, AdrianaGotteland, M.Duttaroy, AssimFrazier, ElisabethGoulet, EricEchevarria, EnriqueFreake, Hedley C.Gouni-Berthold, IoannaEdwards, Christine A.Freedman, MarjorieGoya, LuisEgido, JesusFrei, BalzGrant, Melissa M.Elad, SharonFriis, HenrikGrant, William B.Elli, LucaFriso, SimonettaGreen, Peter H. R.Ellinger, SabineFritsche, KevinGreen, TimEllwood, PhilippaFroessler, BerndGreenberg, Norman A.Eneroth, HannaFu, YangxinGromadzińska, JolantaEngelen, Mariëlle P.K.J.Fu, YingbinGrosso, GiuseppeEnns, CarolineFuglestad, Anita J.Guallar, PilarErbenich, LotharGabrielsson, BrittGugolek, AndrzejGura, KathleenIkeda, YasumasaKasarda, DonGustafson, DeborahImbard, ApollineKaster, ManuellaHaboubi, NadimIngenbleek, YvesKatz, StuartHahm, Eun-RyeongIotti, S.Kaugars, AstridaHahm, Ki-BaikIrvin, VeronicaKawanaka, KentaroHammond, BillyIshimi, Y.Kearney, JohnHan, J. Y.Istfan, NawfalKeenan, MichaelHankey, JoanneJablonska, EwaKeenan, Michael J.Hanna, LynnJackson, Matthew I.Keller, KathleenHarris, MaryJames, JenniferKelly, AlanHarrison, FionaJamshidi, NeemaKemppainen, TarjaHarrison, JonathanJanda, M.Kerver, JeanHatano, TsutomuJanette De Goede, JanetteKhashan, Ali S.Hausman, Dorothy B.Jarvandi, SoghraKhoo, H. H.Hay, WilliamJawadwala, RehanaKhosla, PramodHayes, John E.Jean, GuillaumeKi, Sung HwanHeaney, RobertJeffreys, MonaKimokoti, Ruth W.Heijboer, Annemieke C.Jelski, WojciechKinugawa, S.Heilbronn, LeonieJenmalm, MariaKirkpatrick, NadineHelmerhorst, EvaJeppsson, BengtKishida, TaroHemila, HarriJi, YeweiKispert, Lowell D.Hida, KyokoJin, PengKlek, StanislawHill, KatieJirillo, EmilioKlims-Zacas, DorothyHinckson, Erica A.Johansson, GunnarKnechtle, BeatHjaltadóttir, IngiborgJohnson, ElizabethKneepkens, C. M. FrankHo, Sai YinJohnston, CarolKobayash, HirotoshiHodge, AllisonJones, Andrew M.Konieczka, PawelHoeller, UlrichJones, EricKopel, SherylHolden, RachelJonnalagadda, SatyaKoplin, JenniferHolick, Michael F.Jorde, RolfKormarnytsky, SlavkoHolmen, Turid LingaasJordheim, MonicaKosman, DanielHolmes, GeoffreyJornayvaz, F. R.Kreichauf, SusanneHornstra, GerardJoung, HyojeeKrishnan, KoyamangalathHoughton, Lisa A.Joven, JorgeKrol, EwelinaHouston, Mark C.Jung, SeungyounKrueger, ThiloHruby, AdelaKabbani, Toufic A.Krumbiegel, DoreenHsieh, Yih-shouKagawa, MasaharuKull, IngerHsing, C.-H.Kang, JongKunz, WolframHsu, Chin-linKang, JonghoonKuro-O, MakotoHuang, DejianKantartzis, KonstantinosKurz, TinoIbiebele, Torukiri I.Kantor, Elizabeth D.Kuzma, ElaIgnacimuthu, SavarimuthuKapur, SanjayLa Gamma, Edmund F.Lacetera, NicolaLi, Yan ChunMantzioris, EvangelineLadas, ElenaLiem, GieMares, JulieLadberg, RikardLin, JenniferMariotti, FrançoisLagerstrom, SusanneLin, Pao-hwaMartens, EricLaing, William A.Lin, Shyh-HsiangMartín-Cordero, CarmenLam, Yan Y.Lin, YongMartin, François-Pierre J.Lambert, Joshua D.Lind, MariaMartin, HarryLandrier, Jean-françoisLinden, JoelMartinez, IciarLane, DariusLiu, David T. L.Maruvada, PadmaLangie, SabineLiu, ZhehuaMascitelli, L.Lanou, Amy JoyLocatelli, FrancescoMasmoudi, OlfaLanzini, AlbertoLodge, JohnMasso-Welch, Patricia A.Laparra, MoisésLodha, SaileshMastro, AndreaLarsen, Bodil M. K.Loizzo, Monica R.Masuzaki, HiroakiLarsen, Vanessa GarciaLokeshwar, BalakrishnaMatarese, GiuseppeLarson-Meyer, D. EnetteLoprinzi, Paul D.Matés, José M.Larson, CharlesLores Aguin, MartaMatsuda, RyoichiLattimer, James M.Lucia, DanteMay, James M.Latunde-Dada, Gladys O.Lucock, MarkMayer, GertLaugier, ReneLudvigsson, Jonas F.Mayne, SusanLawen, AlfonsLukowski, Angela F.Mazurak, VeraLayman, DonaldLundin, KnutMcCarthy, DavidLazarus, John H.Luo, Jian-HuaMcCarty, MarkLazzerini, M.Lyon, GrahamMclean, Judy A.Leaf, DavidM, Sarah L. OrganMcclung, JamesLebwohl, BenjaminMa, DavidMcdougall, Gordon J.Lecerf, Jean-michelMaalouf, JoyceMcGrice, Melanie A.Lee, Jeong-chaeMabalirajan, MabalirajanMckinley, MichelleLee, RpMabusela, WilfredMcLeod, RogerLehucher-Michel, Marie-PascaleMace, OliverMclntosh, Michael
MacFarlane, Amanda J.Mcnally, DayreLeibowitz, Sarah F.Machado, MarcoMcnamara, Robert K.Lemos, Manuel C.Magarey, AntheaMcqualter, JonathanLems, WillemMagkos, FaidonMcsharry, CharlesLeon, Gloria R.Magnani, MauroMeans, Robert T.Leung, AngelaMaheshwari, AkhilMedlock, AmyLever, MichaelMaiani, GiuseppeMeinders, A.Levin, AdeeraMakharia, Govind K.Mejia, Elvira GonzalezLewis, JoshMalaguarnera, MicheleMeli, R.Li, Gui-RongMalhotra, AseemMeredith, M. ElizabethLi, KuanrongManrique, DavidMerrill, Rebecca D.Li, MuMantovani, AlbertoMetzler-Zebeli, BarbaraMhurchu, NiNewburg, DavidOtani, HajimeMichels, Alexander J.Nexo, EbbaOteiza, Patricia I.Midgley, AdrianNg, DominicOwusu-Apenten, RichardMiles, Mary P.Ng, Philip C. H.Oz, HeliehMillen, AmyNg, Tze PinPaik, JisunMiller, BenjaminNicklas, Theresa A.Paluszczak, JarosławMiller, Joshua W.Nicol, ChristopherPanahi, ShirinMladěnka, PřemyslNielsen, ForrestPanfoli, IsabellaMøller, PeterNieman, David C.Papoutsakis, ConstantinaMolto, MariaNiemeier, Brandi S.Parinandi, Narasimham L.Monjardino, TeresaNieminen, PetteriPark, Yoon JungMontagne, L.Nikolajczyk, B. S.Park, YoonjungMoon, Hyun-KyungNikolakopoulou, ZacharoulaParker, HeatherMoon, RebeccaNiu, KaijunParnell, Jill A.Moore, AideenNoakes, Paul S.Parthasarathy, SampathMoore, RandleNoland, RobertPassamonti, SabinaMoosmann, BerndNonn, L.Patelarou, EvridikiMoran, NancyNoordzij, M.Payne, MarthaMori, NaokiNorris, Keith C.Pearce, E. N.Morris, HowardNorton, RosemaryPedraza-Chaverri, JoséMoya, FernandoNowson, CarylPena-Rosas, Juan PabloMujico, Jorge R.Nugroho, Rudy AgungPeña, SalvadorMukhopadhyay, ParthaNurmatov, UlugbekPereira, DoraMullen, BillNwosu, Benjamin UdokaPerez, AntonioMumford, Sunni L.Nyakeriga, Alice M.Peterson, Catherine A.Muñoz, ManuelNygård, OttarPeterson, JuliaMurphy, CaoileannO'Brien, PeterPhillips, CatherineMurray, IanO'neal, EricPiacentini, Maria FrancescaMurray, Joseph A.O'neil, CarolPilz, StefanMursu, JaakkoObana, A.Pina, RiccardoMutti, ElenaObeid, RimaPissios, PavlosMuyderman, HakanOberhelman, SaraPoel, Y. H. M.Nagao, AkihikoOdegaard, AndrewPolakof, SergioNaso, AgostinoOgan, DanaPolesel, JerryNathalie M. ScheersOhara, YukiPorter, Judi A.Navarrete, José M. MorenoOkello, EdPosovszky, CarstenNayak, BalunkeswarOlek, Rovert A.Power, Thomas G.Neale, Rachel E.Ollberding, Nicholas J.Powers, Margaret A.Nemeth, ElizabetaOrfila, C.Pownall, HenryNenna, RaffaellaOrmsbee, MichaelPrabhu, K. SandeepNetticadan, ThomasOrton, C. ChristinePrieto-Hontoria, PedroNewbold, BruceOsanai, T.Pritchard, JanetProsser, ColinRossi, MauroSchafer, MarkusPuntis, JohnRostami, KamranSchertzer, Jonathan D.Qadri, FirdausiRoth, DanielSchinke, T.Qi, YanfengRothenberg, Marc E.Schlegel, AmnonQuadros, Edward V.Rottier, BartSchomburg, LutzQureshi, Irfan A.Roubelakis-Angelakis, K. A.Schöttker, BenRacanicci, Aline M. C.Rouvinen-Watt, KirstiSchröder, HelmutRachek, LyudmilaRowland, NeilSchupp, MichaelRaijmakers, N. J. H.Rozanowska, MalgorzataSchurgers, L. J.Rajakumar, KumaravelRozen, RimaSchwab, UrsulaRalston, Nicholas V. C.Rucklidge, JuliaSchwartz, Gary G.Ramos, SoniaRuiz, María DoloresSchwartz, JaniceRandall-Simpson, JanisRupasinghe, H.P. VasanthaSchwarz, Steven M.Ranil, JayawardenaRutsch, FrankSchweigert, FlorianRao, P. VisweswaraRuusunen, AnuSchweitzer, DietrichRavikumara, MadhurRuxto, CarrieScott, HayleyRees, BillRymer, CarolineScott, M. E.Regulska-Ilow, BożenaSacks, GordonSelhub, JacobReichrath, JörgSahibzada, Fayaz AhmadSen, UtpalReid, IanSainaghi, Pier PaoloSennino, BarbaraReis, JoannaSajeva, MaurizioSerra-Majem, LluísReis, Mariza G.Sakhaee, KhashayarSeth, AnjuRicher, Stuart P.Salacinski, A. J.Severance, Emily G.Ried, KarinSaliba, Anthony J.Sharma, NaveenRigo, JacquesSalmi, HeliShay, Kate PetersenRivella, StephanoSalmon, HenriShearer, Gregory C.Rizvi, Ali A.Salvati, SerafinaShih, Mei-FenRoberts, RichardSamman, SamirShin, Bo-ChulRobertson, M. DeniseSánchez, María-JoséSilano, MarcoRobien, KimSanda-Brantland, SolfridSiliquini, RobertaRodriguez-Mateos, AnaSandberg, Ann-SofieSilver, Heidi J.Roma, Eleftheria S.Sanders, DavidSimopoulos, ArtemisRombouts, IneSanders, KerrieSinger, PierreRomesser, Paul B.Sato, MayumiSinha, RaghuRoot, MartinSatokari, ReettaSkeaff, SheilaRoper, Randall J.sauerwein, HelgaSkillman, HeatherRosa, AngeloSavini, IsabellaSkypala, IsabelRose, DevinSaw, Constance Lay LaySlow, SandyRoseman, Mary G.Scaglione, FrancescoSmirnoff, NicholasRosenkranz, SaraScalabrino, GiuseppeSmith, AllenRoss, AlastairScaramuzza, Andrea E.Smithers, LisaRossi, MartaSchaevitz, LauraSmyth, PeterSöderholm, P.Takeda, KiyoshiVan Son, Gabriëlle E.Söderling, Eva M.Tako, EladVaquero, M. P.Sofi, FrancescoTalegawkar, Sameera A.Verger, Eric O.Solomons, NoelTanaka, KiyoshiVermeer, C.Sommer, AlfredTanaka, ToshioVerrill, L.Song, WonTangney, Christine C.Villegas, RaquelSoni, MayaTanguy, StéphaneVincentini, OlimpiaSonia, de Pascual-TeresaTappy, LucVirgilli, FabioSood, AkshayThomas, Lynn K.Virshup, DavidSouberbielle, Jean-ClaudeThomas, SusanVishwanathan, RohiniSowers, James R.Thompson, NicholaVisioli, FrancescoSpeckmann, BodoTinker, Sarah C.Visser, MarliekeStadlbauer, V.Tipoe, George L.Vogel, SilkeStahl, ChadTissing, WimVolpe, StellaStathopoulos, CostasToepel, Ulrikevon Lintig, JohannesStea, TonjeTognon, Gianlucavon Wettstein, D.Stefanidou, Maria E.Tomaino, KatherineVormann, JürgenSteffen, LynTorring, OveVorsa, NicholiStehouwer, Coen D. A.Tosi, PaolaVan Wagoner, David R.Stepto, Nigel K.Trabulsi, JillianWakabayashi, IchiroSteve, Pratt MdTraglia, MichelaWalker, Christa L. FischerStevens, RebeccaTremblay, AngeloWall, Benjamin T.Steves, Claire JoanneTripolt, NorbertWallace, DanielStewart, MariaTrovato, G.M.Walter, Michael H.Stohs, SidTsao, FrancisWang, Jie JinStrand, Tor A.Tselepis, Alexandros D.Warolin, Joshua PaulStranges, SaverioTsuda, TakanoriWatanabe, HirokoStratos, IoannisTsuji, PetraWatanabe, KeikoSu, Shu-JemTuchman, MendelWatkins, Colleen MarieSu, XiaoTung, Tao-HsinWatters, CorileeSubramanian, S.Tursi, AntonioWatts, Gerald F.Sudano, IsabellaTussing-Humphreys, LisaWax, BenjaminSugiura, MinoruTye-Din, JasonWeaver, ConnieSuh, Hyung JooTylavsky, Frances A.Wehr, ElisabethSuliburska, JoannaUehara, M.Weichhart, ThomasSun, Hui SunUllrich, ReinerWeickert, Martin O.Sundström, PeterUrban, ConstantinWeickert, MartinSunkara, LakshmiVachali, PreejithWeiss, NorbertSuratt, BenjaminValenti, PieraWelsh, PaulSuzutani, TatsuoValitutti, FrancescoWhite, JohnTaegtmeyer, HeinrichVallvé, Joan-CarlesWhited, Matthew C.Takaya, JunjiVan Der Kamp, Jan WillemWhiteley, PaulWhiting, SusanWouters, E. F. M.Zeevalk, G. D.Wieringa, Frank T.Wright, BerniceZgaga, LinaWiesenfeld, PaddyWu, JianpingZhang, De-LiangWilliams, ElizabethWylie-Rosett, JudithZhang, LiWilliams, LyndaXian, Cory J.Zhang, MengliangWils, DanielXun, PengchengZhao, Lan-JuanWilson, JohnYakes, ElizabethZhu, ChangfuWilson, Philip K.Yamashita, YoshihisaZhu, KathyWilson, TedYamazaki, TomomiZiberna, LovroWingren, Carl JohanYasuko, ManabeZiouzenkova, OulianaWisniewska-Becker, AnnaYe, XibiaoZittermann, ArminWolfl, C.Yokoyama, YoshihitoZou, SigeWong, W. S. FredYoshida, MunehikoZwart, Sara RWoodside, J. V.Zarogoulidis, Paul


